# Inteligencia artificial y atención primaria: entre la adaptación y la pérdida de centralidad

**DOI:** 10.1016/j.aprim.2026.103556

**Published:** 2026-07-02

**Authors:** Claudia Escoté Llopis, Núria Barrera

**Affiliations:** a Facultat de Medicina i Ciències de la Salut, Universitat de Barcelona, Barcelona, España; b Servicio de Medicina Interna, Hospital Universitari Sagrat Cor, Grupo Quirónsalud, Barcelona, España

*Sr. Editor:*

He leído con gran interés el artículo «Inteligencia artificial y desinformación en salud: la necesidad de reeducación desde la atención primaria». Quisiera felicitar a los autores por ofrecer una reflexión actual y necesaria sobre el papel de la inteligencia artificial (IA) en la generación y difusión de desinformación en salud, así como poner en valor la función educativa de la atención primaria.

El artículo describe de forma acertada tanto los beneficios potenciales de la IA como los riesgos asociados a su uso indiscriminado, especialmente en un contexto en que la información sanitaria es cada vez más accesible, pero no siempre fiable. En este sentido, el papel de los profesionales de atención primaria como agentes de reeducación y filtrado crítico resulta fundamental^1^.

Asimismo, esta propuesta plantea una cuestión relevante: ¿hasta qué punto la atención primaria mantiene hoy su posición central como referente asistencial e informativo para la población? En los últimos años, se ha observado una progresiva transformación en los patrones de acceso al sistema sanitario, con un incremento del uso de urgencias, consultas privadas y, especialmente, fuentes digitales de información^2^, ^3^, incluso aquellas basadas en la inteligencia artificial. Este fenómeno puede estar contribuyendo a una cierta pérdida de centralidad de la atención primaria, que tradicionalmente había sido la puerta de entrada y el principal espacio de confianza para los pacientes^2^.

Esta realidad puede limitar la capacidad de la atención primaria para ejercer un rol educativo, tal como los autores proponen. Si los pacientes consultan antes otras fuentes, muchas veces digitales y no siempre rigurosas^2^, ^3^, la intervención del profesional sanitario podría llegar tarde en un contexto de creencias ya consolidadas. Por lo tanto, la reeducación no solo debería producirse en la consulta, sino también en los mismos entornos digitales donde se genera y se difunde la desinformación.

En este sentido, podría ser necesario complementar el papel tradicional de la atención primaria con estrategias más proactivas^4^, como la presencia institucional en plataformas digitales, la generación de contenido fiable y accesible o la colaboración con desarrolladores de herramientas de IA para garantizar la calidad de la información proporcionada. Esto implicaría un cambio de paradigma, pasando de un modelo reactivo a un modelo más preventivo y adaptado a los nuevos canales y medios de comunicación.

En conclusión, aunque la atención primaria continúa siendo un pilar fundamental del sistema sanitario, su capacidad para actuar como agente de reeducación en la era de la IA dependerá de su adaptación a un entorno informativo en constante evolución. Reconocer esta realidad es esencial para poder reforzar su papel y garantizar una respuesta efectiva ante la desinformación en salud.

## Autoría

Claudia Escoté Llopis ha concebido, redactado y revisado el manuscrito junto a Núria Barrera, y ambas aprueban la versión final enviada.

## Consentimiento informado

No aplicable, al tratarse de una carta al director, no contiene datos procedentes de estudios realizados en pacientes ni en animales por ninguno de los autores.

## Consideraciones éticas

No aplicable, al ser una carta al director que no contiene datos de estudios con pacientes ni animales.

## Uso de IA

Durante la redacción de este manuscrito la autora usó la IA Claude (Anthropic) únicamente con el fin de corregir la ortografía y gramática. Tras esta corrección la autora revisó el contenido y asume la responsabilidad total del texto.

## Conflicto de intereses

Las autoras declaran no tener ningún conflicto de intereses.

## Financiación

Este trabajo no ha recibido ningún tipo de financiación.
